# Structure of human farnesyl pyrophosphate synthase in complex with an aminopyridine bisphosphonate and two molecules of inorganic phosphate

**DOI:** 10.1107/S2053230X14002106

**Published:** 2014-02-19

**Authors:** Jaeok Park, Yih-Shyan Lin, Youla S. Tsantrizos, Albert M. Berghuis

**Affiliations:** a Department of Biochemistry, McGill University, 3655 Promenade Sir William Osler, Montreal, QC H3G 1Y6, Canada; b Department of Chemistry, McGill University, 801 Rue Sherbrooke Ouest, Montreal, QC H3A 0B8, Canada; c Groupe de Recherche Axé sur la Structure des Protéines, McGill University, 3649 Promenade Sir William Osler, Montreal, QC H3G 0B1, Canada; d Department of Microbiology and Immunology, McGill University, 3775 Rue University, Montreal, QC H3A 2B4, Canada

**Keywords:** farnesyl pyrophosphate synthase, bisphosphonates, inorganic phosphate, anomalous diffraction

## Abstract

Human farnesyl pyrophosphate synthase (hFPPS) produces farnesyl pyrophos­phate, an isoprenoid essential for a variety of cellular processes. The enzyme has been well established as the molecular target of the nitrogen-containing bisphosphonates (N-BPs), which are best known for their antiresorptive effects in bone but are also known for their anticancer properties. Crystal structures of hFPPS in ternary complexes with a novel bisphosphonate, YS0470, and the secondary ligands inorganic phosphate (P_i_), inorganic pyrophosphate (PP_i_) and isopentenyl pyrophosphate (IPP) have recently been reported. Only the co-binding of the bisphosphonate with either PP_i_ or IPP resulted in the full closure of the C-­terminal tail of the enzyme, a conformational change that is required for catalysis and that is also responsible for the potent *in vivo* efficacy of N-BPs. In the present communication, a co-crystal structure of hFPPS in complex with YS0470 and two molecules of P_i_ is reported. The unusually close proximity between these ligands, which was confirmed by anomalous diffraction data, suggests that they interact with one another, with their anionic charges neutralized in their bound state. The structure also showed the tail of the enzyme to be fully disordered, indicating that simultaneous binding of two P_i_ molecules with a bisphosphonate cannot induce the tail-closing conformational change in hFPPS. Examination of homologous FPPSs suggested that this ligand-dependent tail closure is only conserved in the mammalian proteins. The prevalence of P_i_-bound hFPPS structures in the PDB raises a question regarding the *in vivo* relevance of P_i_ binding to the function of the enzyme.

## Abstract 2

A co-crystal structure of human farnesyl pyrophosphate synthase in complex with an aminopyridine bisphosphonate, YS0470, and two molecules of inorganic phosphate has been determined. The identity of the phosphate ligands was confirmed by anomalous diffraction data.

## 1. Introduction   

Human farnesyl pyrophosphate synthase (hFPPS) occupies the first branching point in the mevalonate pathway and carries out the elongation of dimethylallyl pyrophosphate (DMAPP) to geranyl pyrophosphate (GPP) and then to farnesyl pyrophosphate (FPP) by successively condensing two molecules of isopentenyl pyrophosphate (IPP). FPP is absolutely required for post-translational modification (*i.e.* prenylation) of small signalling GTPases, which is essential for their subcellular localization and function (McTaggart, 2006 ▶). Given the role of small GTPases as regulators of fundamental cellular processes, blocking their prenylation *via* hFPPS inhibition serves as a useful and effective means of pharmacological intervention. Currently, nitrogen-containing bisphosphonates (N-BPs), such as zoledronate and risedronate, comprise the only class of clinically approved drugs targeting hFPPS. These drugs have been widely used against bone-resorption disorders, but are also gaining a great deal of interest for their anticancer properties (Koul *et al.*, 2012 ▶).

The mechanism by which N-BPs inhibit hFPPS has been well characterized by X-ray crystallographic studies (Kavanagh *et al.*, 2006 ▶; Rondeau *et al.*, 2006 ▶). They bind to the DMAPP/GPP sub­pocket of the active site, mimicking and competing with these substrates. The inhibition also involves ligand-induced conformational changes in the enzyme. Occupancy of the DMAPP/GPP subpocket results in a rigid-body movement that closes this binding site and shapes the IPP subpocket (*i.e.* from the open to the partially closed state). Subsequent IPP binding induces full structuring of the four-residue C-terminal tail, which in turn closes the IPP subpocket and sequesters the active site from the solvent environment (*i.e.* from the partially closed to the fully closed state). While hFPPS cycles through these conformational changes during catalysis, with chemically stable N-BPs the ternary enzyme complex becomes locked in the fully closed state. In this conformation, direct competition between the deeply bound inhibitor and DMAPP/GPP is impossible, and thus N-BP binding is considered to be nearly irreversible. The potent *in vivo* efficacy of N-BP drugs is therefore thought to arise in part from the stabilization of the enzyme–inhibitor complex by binding of the accumulating substrate IPP.

Despite the importance of the C-terminal tail closure in hFPPS, the mechanistic details of this conformational change have remained largely uncharacterized. By determining and analyzing crystal structures of hFPPS in ternary complexes with a novel bisphosphonate inhibitor, YS0470, and the secondary ligands inorganic phosphate (P_i_), inorganic pyrophosphate (PP_i_) and IPP, we recently identified the key residues and interactions responsible for the tail closure of the enzyme (Park *et al.*, 2012 ▶). The secondary ligands were introduced by soaking in this study, and only the binding of PP_i_ or IPP induced the full structuring of the C-terminal tail of the enzyme. More recently, we solved a crystal structure of hFPPS obtained under different conditions that exhibited unusual electron density in the second substrate-binding site. Analysis of anomalous diffraction data from an isomorphous crystal has allowed us to unambiguously identify the ligands bound at this site. We thus report in this communication a co-crystal structure of hFPPS in complex with YS0470 and two molecules of P_i_.

## 2. Materials and methods   

### 2.1. Preparation of the protein and inhibitor samples   

The expression and purification of hFPPS, as well as the synthesis of YS0470, have been described in a previous report (Lin *et al.*, 2012 ▶).

### 2.2. Crystallization   

Compound YS0470 was prepared as a 100 m*M* solution in 100 m*M* Tris–HCl pH 7.5, and MgCl_2_ was prepared as a 100 m*M* aqueous solution. These solutions were added to the hFPPS sample to give final concentrations of 1 m*M* inhibitor, 1.5 m*M* MgCl_2_ and 0.25 m*M* (10 mg ml^−1^) protein. Crystals suitable for X-ray diffraction were obtained at 295 K by vapour diffusion in a sitting drop composed of 1 µl inhibitor/MgCl_2_/protein mixture, 1 µl crystallization solution (30% PEG 400, 2 *M* ammonium phosphate, 0.2 *M* MgCl_2_, 0.1 *M* HEPES pH 7.5) and 0.5 µl seed stock. The seed stock was prepared with a Seed Bead kit (Hampton Research) using a crystal grown in a sitting drop consisting of 1 µl ligand-free protein sample (10 mg ml^−1^) and 1 µl crystallization solution (2.0 *M* ammonium phosphate, 0.1 *M* Tris–HCl pH 8.5).

### 2.3. Data collection, processing and structure refinement   

For structure determination, diffraction data were collected from a single crystal at 100 K using synchrotron radiation (Canadian Light Source, Saskatoon, SK, Canada) and a Rayonix MX300 CCD detector. For exploiting the anomalous signal from P atoms, additional data were collected at home from another single crystal using a MicroMax-007 HF generator (Rigaku) and a Saturn 944+ CCD detector (Rigaku). Both data sets were processed with the *xia*2 package (Winter *et al.*, 2013 ▶); for the home-source data the Friedel mates were not merged together, unlike for the synchrotron data. The structure model was initially built by a difference Fourier method with a ligand/solvent-omitted starting model generated from PDB entry 4h5d (Park *et al.*, 2012 ▶). The model was improved through iterative rounds of manual and automated refinement with *Coot* (Emsley *et al.*, 2010 ▶) and *REFMAC*5 (Murshudov *et al.*, 2011 ▶). The final model was deposited in the Protein Data Bank (PDB entry 4lfv). Data-collection and refinement statistics are presented in Table 1 ▶.

### 2.4. Anomalous density calculation   

An anomalous density map was calculated from the home-source data with the programs *SHELXC* (Sheldrick, 2010 ▶) and *ANODE* (Thorn & Sheldrick, 2011 ▶). The phase information used in this calculation was obtained from the final structure model refined against the synchrotron data.

## 3. Results and discussion   

### 3.1. Overall structure   

The overall fold of the new structure (PDB entry 4lfv) is very similar to those of the previously described hFPPS–YS0470 complexes, as indicated by the r.m.s.d.s for superposition in Table 2 ▶. It is noteworthy that the r.m.s.d. values are lower with the secondary ligand-free (hFPPS–YS0470) and P_i_-bound (hFPPS–YS0470–P_i_) forms, which are in the partially closed conformation. This observation is consistent with the finding that the new complex is also in the partially closed conformation, the details of which we will discuss below.

### 3.2. DMAPP/GPP subpocket and bisphosphonate binding   

The structure of the DMAPP/GPP subpocket is essentially identical in all of the reported YS0470-bound hFPPS complexes, including the present one. The interactions between the bisphosphonate and the protein have been discussed previously (De Schutter *et al.*, 2012 ▶; Lin *et al.*, 2012 ▶; Park *et al.*, 2012 ▶).

### 3.3. IPP subpocket and secondary ligands   

**Figure 1:**
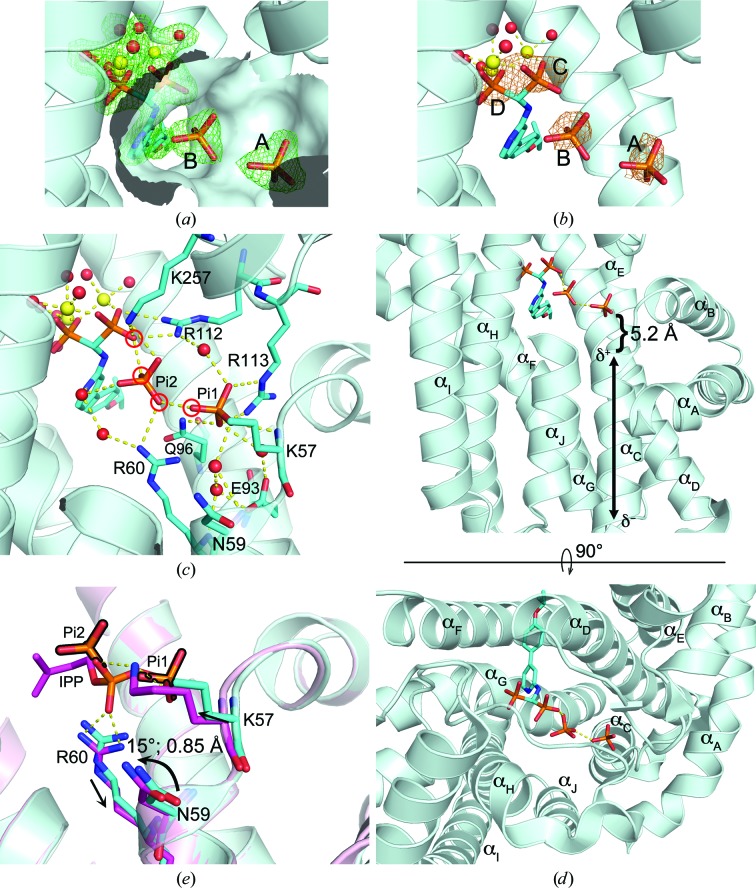
Ligand binding in the hFPPS–YS0470–2P_i_ complex. (*a*) The initial Fourier synthesis map (green mesh, *F*_o_ − *F*_c_, contoured at 3σ) showing the electron densities for the bound ligands (sticks) and the metal (yellow spheres) coordinated water molecules (red spheres). The protein surface within 4 Å radius of the bound P_i_ molecules is shown to indicate the IPP subpocket. (*b*) An anomalous Fourier map (orange mesh, contoured at 3σ) superimposed onto the structure model. The heights of the anomalous peaks were 4.9, 4.9, 6.1 and 7.8σ for A, B, C and D, respectively. (*c*) Interactions between the bound ligands, water molecules and the residues of the IPP subpocket. Note that the side chain of Lys57 could not be fully modelled owing to disorder. (*d*) Secondary-structure elements around the bound ligands. The nomenclature follows that of Tarshis *et al.* (1994 ▶). The dipole of the relevant helix is shown. (*e*) Superposition of the hFPPS–YS0470–2P_i_ complex and the hFPPS–YS0470–IPP complex (PDB entry 4h5e, magenta) at the IPP subpocket. The two P_i_ molecules are outlined in black. Note that P_i_1 superposes with the terminal phosphate of IPP (and also with the single P_i_ bound in the hFPPS–YS0470–P_i_ complex; not shown).

The initial density map produced by Fourier synthesis, phased with only protein atoms, indicated the presence of two ligands in the IPP subpocket. We could readily deduce the identity of one (density A in Fig. 1 ▶ *a*) as P_i_ based on the shape of its electron density as well as its location as a known P_i_-binding site (Park *et al.*, 2012 ▶). The most likely candidate for the second ligand was also P_i_, given the electron-density contour (density B in Fig. 1 ▶ *a*) and the composition of the crystallization mother liquor (see §[Sec sec2.2]2.2). However, the proximity of the second P_i_ to both the first P_i_ and the bisphosphonate group of YS0470 (closest atomic distances of 2.3 and 2.5 Å, respectively) was puzzling, since all three of these ions should be negatively charged at the given pH and thus be subject to electrostatic repulsion.

In order to verify the identity of the second P_i_, we collected a second data set in which the anomalous signal was preserved during processing. With Cu *K*α radiation (λ = 1.5418 Å) P atoms displayed a measurable anomalous signal: the anomalous scattering contribution (*f*′′) of phosphorus is appreciable at this wavelength (0.45 electron units), although its X-ray absorption edge lies at a much longer wavelength (λ = 5.7788 Å; Brennan & Cowan, 1992 ▶). As a result, an anomalous Fourier map calculated from the anomalous data set clearly demonstrated peaks that superposed on the P atoms of the first P_i_ and YS0470 in our structure model, as well as that of the second P_i_, thus confirming its identity (Fig. 1 ▶ *b*).

The close proximity between the bisphosphonate and phosphate ligands suggests that their molecular charges may be neutralized by the surrounding ions and protons either on the nearby residues or the ligands. In addition to three Mg ions chelated to the bisphosphonate, the residues Arg60, Arg112, Arg113 and Lys257 are likely contributors, forming direct contacts with these ligands (Fig. 1 ▶ *c*). Furthermore, the helix dipole of α_C_ may also play a role in this regard, dissipating some of the charge on the first P_i_ (Fig. 1 ▶ *d*). Phosphate moieties frequently bind to the amino-termini of protein helices, typically at a distance of 3–5 Å, owing to the electric field generated by the helix backbone (Hol *et al.*, 1978 ▶). Although our crystallographic data do not provide information regarding the protonation state of the ligands, the geometry and distances between the four interacting O atoms of the bisphosphonate and P_i_ (circled in red in Fig. 1 ▶ *c*) suggest that they form hydrogen bonds, with two of them being protonated.

As mentioned above, the enzyme–ligand complex reported here (hFPPS–YS0470–2P_i_) is in the partially closed state, like the hFPPS–YS0470 and hFPPS–YS0470–P_i_ complexes. The conformational difference between the partially closed state and the fully closed state (as observed with the hFPPS–YS0470–PP_i_ and hFPPS–YS0470–IPP complexes) is not overtly pronounced in the IPP subpocket: the α-­phosphate of the bound IPP (or the equivalent phosphate of the PP_i_) attracts and structures Lys57 while pushing back Arg60; the displacements of these residues by less than 1 Å result in a ∼15° rotation of Asn59 (Fig. 1 ▶ *e*). The second P_i_ in the new complex, in comparison, is bound too distantly to interact with Lys57 (P_i_2; Fig. 1 ▶ *e*) and thus cannot bring about the above conformational changes. Although subtle, these changes are solely responsible for the subsequent ordering and closing of the C-terminal tail in hFPPS.

### 3.4. C-terminal tail closure   

**Figure 2:**
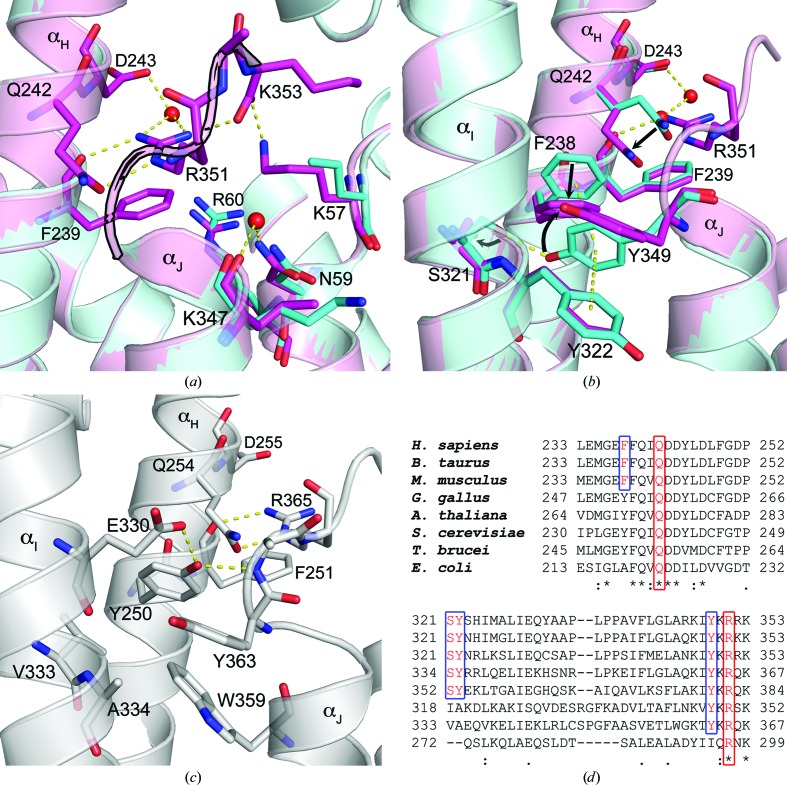
Residues of the FPPS tail closure. (*a*) Tail closure induced by IPP binding. The hFPPS–YS0470–2P_i_ complex (cyan) and the hFPPS–YS0470–IPP complex (magenta) are superposed, with the bound ligands omitted for clarity. Note that the hFPPS–YS0470–2P_i_ complex is missing the C-terminal tail, which in the hFPPS–YS0470–IPP complex (highlighted in black) closes over the active-site entrance. The structures are shown in approximately the same orientation as in Fig. 1 ▶(*e*) (compare residues Lys57, Asn59 and Arg60). (*b*) The conformational change cascade required for the tail closure. In the absence of bound IPP/PP_i_, Tyr349 is trapped in the ‘off’ conformation by π-stacking and hydrogen-bond interactions. The ‘off’ conformations of Tyr349, Phe238 and Gln242 (cyan) prohibit the ‘on’ conformations of Phe238, Gln242 and Arg351 (magenta), respectively, by steric hindrance. (*c*) A second ligand-free *T. brucei* FPPS complex (PDB entry 3dyh, white) with its ordered C-terminal tail. (*d*) Conserved residues of the FPPS tail closure. Sequence alignment was carried out with *ClustalX* (Larkin *et al.*, 2007 ▶).

Previously, we observed electron density suggesting partial ordering of the C-terminal tail in the hFPPS–YS0470 and hFPPS–YS0470–P_i_ complexes, and could refine only the backbone atoms of the tail in the structure models (Lin *et al.*, 2012 ▶; Park *et al.*, 2012 ▶). The corresponding electron density was weaker for the hFPPS–YS0470–2P_i_ complex, and the four C-terminal residues could not be built into our new structure model. In contrast, the C-terminal tail in the hFPPS–YS0470-PP_i_ and hFPPS–YS0470–IPP complexes was shown to be fully structured (Park *et al.*, 2012 ▶). The full structuring of the C-­terminal tail is most notably characterized by ordering of the Arg351 side chain, which anchors itself to helix α_H_ and also forms a salt bridge with the terminal residue Lys353, thereby providing the otherwise flexible tail with rigidity (Fig. 2 ▶ *a*).

We also showed previously that the anchoring of Arg351 requires a series of preceding conformational changes in the residues Gln242, Phe238 and Tyr349 (Fig. 2 ▶ *b*; Park *et al.*, 2012 ▶). Tyr349 is likely to function as a safety switch to prevent futile tail closure in the absence of bound IPP: when it is locked in the ‘off’ position, the downstream conformational changes are prohibited by steric hindrance (Fig. 2 ▶ *b*). Examination of homologous FPPS proteins provides interesting perspectives regarding this control mechanism. In *Trypanosoma brucei* FPPS, Tyr363 cannot assume the ‘off’ conformation observed for its human counterpart owing to a neighbouring tryptophan (Trp359) hindering such a conformation (Fig. 2 ▶ *c*). In addition, Tyr250, which corresponds to Phe238 in the human enzyme, is trapped in the ‘on’ conformation by two adjacent residues *via* hydrogen bonds (Fig. 2 ▶ *c*). Not surprisingly, the C-terminal tail of *T. brucei* FPPS is fully structured in all nine structures available in the PDB, regardless of the presence of bound IPP (*e.g.* PDB entry 3dyh; Fig. 2 ▶ *c*; see Supplementary Table S1^1^ for a complete list). *T. cruzi* FPPS also shows similar conformations for the equivalent residues and the C-­terminal tail (12 structures in the PDB; Supplementary Table S1), although it has a histidine residue at the position of the switch tyrosine. Interestingly, the switch tyrosine and the three interacting residues (*i.e.* Phe238, Ser321 and Tyr322 in hFPPS) are fully conserved only in mammalian species, in contrast to the indispensable glutamine and arginine residues (Fig. 2 ▶ *d*). This observation suggests that the tail-closure control feature in FPPS is exclusively characteristic of the mammalian order. A putative mechanism by which the subtle IPP/PP_i_-induced conformational change in the IPP subpocket is translated into such a drastic movement of Tyr349 (*i.e.* >70° rotation of the side chain), thus allowing the full closure of the C-terminal tail in hFPPS, has been described in detail previously (Park *et al.*, 2012 ▶).

### 3.5. Significance of P_i_ binding   

A retrospective examination of all of the previously determined structures of hFPPS in the PDB (30 entries, excluding our own entries) identified 19 structures that have bound P_i_ in the IPP subpocket (Supplementary Table S2). Other non-substrate ligands shown to bind at this site include inorganic pyrophosphate and sulfate. It is presently unknown whether the binding of P_i_ or other negatively charged ions to hFPPS is a physiologically relevant event or strictly a crystallization artefact. However, it is possible that P_i_ binding occurs *in vivo*, especially with its millimolar-scale intracellular concentration (Bevington *et al.*, 1986 ▶). In such a case P_i_ would inhibit the enzyme by competing with the substrate IPP, the intracellular concentration of which, in contrast, is in the picomolar range (Mönkkönen *et al.*, 2008 ▶). Although the kinetics have not been studied in detail, *in vitro* inhibition of FPPS by a high concentration of P_i_ was reported a long time ago (Holloway & Popják, 1967 ▶). Interestingly, elevated levels of P_i_ produce antiproliferative effects in multiple cancer cell lines by an as yet unknown mechanism involving reduced ERK1/2 phosphorylation (Spina, Sapio *et al.*, 2013 ▶; Spina, Sorvillo *et al.*, 2013 ▶). As downregulation of ERK phosphorylation is also a cellular hallmark of hFPPS inhibition (Lin *et al.*, 2012 ▶), it is conceivable that P_i_ elicits the antiproliferative effects (in part) by inhibiting hFPPS. However, this hypothesis is at present unproven.

## 4. Conclusion   

The crystal structure of hFPPS reported in this communication demonstrates that the IPP subpocket of the enzyme can accommodate two molecules of P_i_ simultaneously. The binding of two P_i_ molecules did not induce the C-terminal tail closure in the enzyme, unlike that of IPP or PP_i_. This ligand-controlled conformational change is likely to be conserved only in mammalian FPPS. The possibility that P_i_ serves as a modulator of hFPPS function *in vivo* warrants future studies. Our work also showed that the new program *ANODE* is effective in analyzing weak anomalous data and can be useful in identifying unknown ligands.

## Supplementary Material

PDB reference: farnesyl pyrophosphate synthase, complex with YS0470 and two molecules of inorganic phosphate, 4lfv

Supporting Information.. DOI: 10.1107/S2053230X14002106/hv5250sup1.pdf

## Figures and Tables

**Table 1:** Data-collection and structure-refinement statistics Values in parentheses are for the outer shell.

	Data set 1 (synchrotron)	Data set 2 (home source)
Data collection		
Wavelength ()	0.97949	1.5418
Space group	*P*4_1_2_1_2	*P*4_1_2_1_2
Unit-cell parameters (, )	*a* = *b* = 111.0, *c* = 67.0, = = = 90	*a* = *b* = 111.0, *c* = 69.9, = = = 90
No. of molecules in asymmetric unit	1	1
Matthews coefficient *V*_M_ (^3^Da^1^)	2.39	2.50
Solvent content (%)	48.62	50.74
Resolution ()	67.042.00 (2.052.00)	39.242.46 (2.522.46)
No. of unique reflections	28844 (2083)	16393 (1144)
Mean *I*/(*I*)	28.8 (7.4)	49.2 (3.8)
Completeness (%)	99.7 (98.8)	98.7 (87.5)
Multiplicity	14.3 (14.3)	13.2 (2.7)
*R*_merge_	0.070 (0.498)	0.054 (0.389)†
Structure refinement		
Resolution range ()	51.032.00 (2.052.00)	
No. of reflections used, working set	27134 (1882)	
No. of reflections used, test set	1454 (110)	
No. of protein atoms in the model	2728	
No. of water atoms in the model	153	
No. of other atoms in the model	40	
Overall average *B* factor (^2^)	35.0	
Final *R*_work_	0.177 (0.202)	
Final *R*_free_	0.218 (0.265)	
R.m.s.d., bond lengths ()	0.019	
R.m.s.d., bond angles ()	1.923	
Residues in Ramachandran regions (%)		
Favoured region	98.5	
Allowed region	1.5	
Outlier region	0	

† When merged.

**Table 2:** PDB structures of hFPPS in complex with the bisphosphonate YS0470

PDB code (reference)	Resolution ()	Ligands	R.m.s.d.† ()	Overall conformation
4dem (Lin *et al.*, 2012 ▶)	1.85	YS0470‡	0.17	Partially closed
4h5c (Park *et al.*, 2012 ▶)	2.02	YS0470, P_i_	0.17	Partially closed
4h5d (Park *et al.*, 2012 ▶)	2.02	YS0470, PP_i_	0.29	Fully closed
4h5e (Park *et al.*, 2012 ▶)	2.05	YS0470, IPP	0.25	Fully closed
4lfv (this work)	2.00	YS0470, 2P_i_		Partially closed

† Structure superposition was performed with *SSM* based on 335 C atoms.

‡ Bisphosphonate binding occurs *via* metal chelation involving three Mg ions, which are not included in this table.

## Footnotes

1 Supporting information has been deposited in the IUCr electronic archive (Reference: HV5250).
