# Supplementary material for: Structure of human farnesyl pyrophosphate synthase in complex with an aminopyridine bisphosphonate and two molecules of inorganic phosphate
Source: Acta Crystallogr F Struct Biol Commun. 2014 Feb 19;70(Pt 3):299–304. doi: 10.1107/S2053230X14002106 (PMC3944689; doi:10.1107/S2053230X14002106)
Supplement: Supplementary file 1 [file f-70-00299-sup1.pdf]

Supporting Information

Other PDB structures of FPPS examined in this study

Table S1. PDB structures of protozoan FPPS

Trypanosoma brucei FPPS		*Trypanosoma cruzi* FPPS	
PDB code (literature ref.)	Ligands in IPP pocket	PDB code (ref.)	Ligands in IPP pocket
2ewg (Mao *et al.*, 2006)	none	1yhl (Gabelli *et al.*, 2006)	DMAPP
2i19 (Mao *et al.*, 2006)	none	1yhm (Gabelli *et al.*, 2006)	IPP
2ogd (not available)	two molecules of acetate	3iba (Huang *et al.*, 2010)	IPP, sulfate[b]
2p1c (Cao *et al.*, 2008)	acetate[a]	3ick (Huang *et al.*, 2010)	IPP, sulfate[b]
3dyf (Zhang *et al.*, 2009)	IPP	3icm (Huang *et al.*, 2010)	IPP, sulfate[b]
3dyg (Zhang *et al.*, 2009)	acetate	3icn (Huang *et al.*, 2010)	IPP, sulfate[b]
3dyh (Zhang *et al.*, 2009)	none	3icz (Huang *et al.*, 2010)	IPP
3efq (Zhang *et al.*, 2009)	none	3id0 (Huang *et al.*, 2010)	sulfate
3egt (Zhang *et al.*, 2009)	none	4dwb (Aripirala *et al.*, 2012)	IPP
		4dwg (Aripirala *et al.*, 2012)	sulfate
		4dxj (Aripirala *et al.*, 2012)	IPP
		4e1e (Aripirala *et al.*, 2012)	IPP

[a]There are two molecules of acetate bound in one protomer, and one in the other (two protomers in the asymmetric unit). [b]IPP and sulfate were both modeled in at half occupancy each. Note that the two other *T. cruzi* FPPS structures available in the PDB, 1yhk and 4dzw, were not included here because the former has no bound bisphosphonate, and the latter is of low resolution (3.05 Å).

Table S2 PDB structures of hFPPS with bound Pi in the IPP sub-pocket

PDB code (literature ref.)	Bisphosphonate ligands	# of Pi
1yq7 (not available)	residronate	2
1yv5 (Kavanagh *et al.*, 2006)	residronate	2
2f7m (Rondeau *et al.*, 2006)	none	1
2f89 (Rondeau *et al.*, 2006)	pamidronate	1
2f8c (Rondeau *et al.*, 2006)	zoledronate	1
2f92 (Rondeau *et al.*, 2006)	alendronate[a]	1
2f94 (Rondeau *et al.*, 2006)	ibandronate[a]	1
2f9k (Rondeau *et al.*, 2006)	zoledronate[a]	1
2opm (Zhang *et al.*, 2009)	3-fluoro-1-(2-hydroxy-2,2-diphosphonoethyl)pyridinium	2
2opn (not available)	[2-(dimethyl-lambda~4~-sulfanyl)-1-hydroxyethane-1,1-diyl]bis(phosphonic acid)	2
3n1v (Jahnke *et al.*, 2010)	none	1
3n1w (Jahnke *et al.*, 2010)	none	1
3n45 (Jahnke *et al.*, 2010)	zoledronate	2
3n46 (Jahnke *et al.*, 2010)	zoledronate	1
3n49 (Jahnke *et al.*, 2010)	none	1
3n5h (Jahnke *et al.*, 2010)	none	1
3n5j (Jahnke *et al.*, 2010)	none	1
3n6k (Jahnke *et al.*, 2010)	none	1
4ga3 (Zhang *et al.*, 2013)	1-butyl-3-(2-hydroxy-2,2-diphosphonoethyl)-1H-imidazol-3-ium	2

[a]Bisphosphonates are bound with Zn ions instead of Mg ions.
